# Potential for imaging the high-affinity state of the 5-HT_1B_ receptor: a comparison of three PET radioligands with differing intrinsic activity

**DOI:** 10.1186/s13550-019-0570-1

**Published:** 2019-11-21

**Authors:** Anton Lindberg, Ryosuke Arakawa, Tsuyoshi Nogami, Sangram Nag, Magnus Schou, Charles S. Elmore, Lars Farde, Victor W. Pike, Christer Halldin

**Affiliations:** 1 Department of Clinical Neuroscience, Center for Psychiatry Research, Karolinska Institutet and Stockholm County Council, SE-17176 Stockholm, Sweden; 2 Molecular Imaging Branch, National Institute of Mental Health, National Institutes of Health, Bethesda, MD 20892-1003 USA; 3 Isotope Chemistry, Early Chemical Development, Pharmaceutical Sciences R&D, AstraZeneca, SE-43250 Göteborg, Sweden; 4 PET Science Centre, Precision Medicine and Genomics, R&D, AstraZeneca, SE-17176 Stockholm, Sweden

**Keywords:** 5-HT_1B_, PET, Radioligand, Agonist, Antagonist

## Abstract

**Background:**

Over the last decade, a few radioligands have been developed for PET imaging of brain 5-HT_1B_ receptors. The 5-HT_1B_ receptor is a G-protein-coupled receptor (GPCR) that exists in two different agonist affinity states. An agonist ligand is expected to be more sensitive towards competition from another agonist, such as endogenous 5-HT, than an antagonist ligand. It is of interest to know whether the intrinsic activity of a PET radioligand for the 5-HT_1B_ receptor impacts on its ability to detect changes in endogenous synaptic 5-HT density. Three high-affinity ^11^C-labeled 5-HT_1B_ PET radioligands with differing intrinsic activity were applied to PET measurements in cynomolgus monkey to evaluate their sensitivity to be displaced within the brain by endogenous 5-HT. For these experiments, fenfluramine was pre-administered at two different doses (1.0 and 5.0 mg/kg, i.v.) to induce synaptic 5-HT release.

**Results:**

A dose-dependent response to fenfluramine was detected for all three radioligands. At the highest dose of fenfluramine (5.0 mg/kg, i.v.), reductions in specific binding in the occipital cortex increased with radioligand agonist efficacy, reaching 61% for [^11^C]**3**. The most antagonistic radioligand showed the lowest reduction in specific binding.

**Conclusions:**

Three 5-HT_1B_ PET radioligands were identified with differing intrinsic activity that could be used in imaging high- and low-affinity states of 5-HT_1B_ receptors using PET. From this limited study, radioligand sensitivity to endogenous 5-HT appears to depend on agonist efficacy. More extensive studies are required to substantiate this suggestion.

## Background

The 5-HT_1B_ receptor is one of 14 serotonin receptor subtypes. This G-protein-coupled receptor (GPCR) has been identified as a potential target for drug treatment of depression and anxiety [1–3]. GPRCs have been shown to exist in at least two different affinity states in vitro, depending on whether the receptor is coupled or uncoupled to the G-protein [4]. Generally, it is thought that agonists bind with higher affinity to the coupled receptor than to the uncoupled receptor and that antagonists bind with equal affinity to both states [5]. The binding of a 5-HT_1B_ receptor agonist or endogenous 5-HT will activate the G-protein and in further steps deactivate the adenylate cyclase coupled to the GPCR. An antagonist can competitively inhibit an agonist or 5-HT from binding to the receptor and an inverse agonist will bind to the receptor and have an inverse effect to an agonist [6]. It is likely that GPCR-ligand interaction is even more complex, and active intermediate states have been suggested [7, 8].

The human 5-HT_1B_ receptor is closely related in structure to the rodent 5-HT_1D_ receptor [9]. In an experimental model proposed for 5-HT binding to the 5-HT_1D_ receptor, 5-HT shows a strong preference for binding to receptors in the G-protein-coupled state. By comparison, an inverse agonist/antagonist (ocaperidone) showed the opposite preference. In addition, [^3^H]5-HT has shown a higher affinity for G-protein-coupled 5-HT_1B_ receptors than for uncoupled receptors in vitro [10].

X-ray crystallography has revealed how ligands of differing intrinsic efficacy at 5-HT_1B_ receptors interact differently with the binding pocket [11, 12]. An agonist binds deeper into the binding pocket than does an inverse agonist or antagonist. It has been proposed that for a ligand to access the deeper part of the binding pocket, the receptor must become activated by binding to a G-protein. A study with positron emission tomography (PET) of the dopamine subtype-2 (D_2_) receptor subtype in non-human primates (NHP) found that an agonist PET radioligand ([^11^C]MNPA) was more sensitive than an antagonist radioligand ([^11^C]raclopride) towards competition from an increased synaptic concentration of endogenous neurotransmitter (i.e., dopamine) induced by the intravenous administration of amphetamine [13]. Taken together, several experimental studies in rodents and in NHPs support the existence of high- and low-affinity states of GCPRs in vivo. In a recent review, Shalgunov et al. describe the various ways high-affinity state receptors can be imaged using PET and the challenges involved [14]. One challenge is the availability of suitable PET radioligands with differing intrinsic activity with high affinity and selectivity towards the same receptor.

**Fig. 1:**
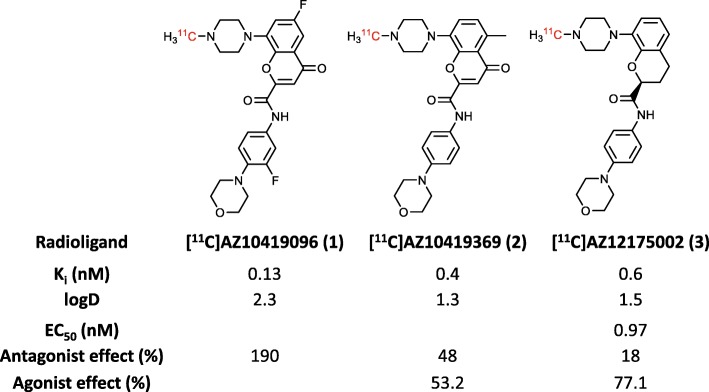
Structures and in vitro data for [^11^C]AZ10419369, [^11^C]AZ10419096, and [^11^C]AZ12175002. Data were provided by AstraZeneca

Three PET radioligands were selected for their differing intrinsic activity. Recently, we reported the development and characterization of a full 5-HT_1B_ receptor antagonist PET radioligand, [^11^C]AZ10419096 ([^11^C]**1**; Fig. 1), for imaging 5-HT_1B_ receptors in NHP brain, and that this radioligand is also sensitive to endogenous 5-HT [15]. [^11^C]AZ10419369 ([^11^C]**2**; Fig. 1) acts as a partial agonist/antagonist at the 5-HT_1B_ receptor. This radioligand demonstrated sensitivity to changes in endogenous 5-HT concentration in previous studies [16]. However, our effort to develop a full agonist radioligand for the 5-HT_1B_ receptor did not succeed, and no such radioligand yet exists [17]. Therefore, we selected the highly agonistic 5-HT_1B_ PET radioligand, ([^11^C]AZ12175002; [^11^C]**3**; Fig. 1), [18] to act as an agonist radioligand for comparison with [^11^C]**1** (antagonist) and [^11^C]**2** (partial agonist/antagonist) in our investigation of the relationship between intrinsic activity and the sensitivity of radioligand receptor binding to changes in the synaptic 5-HT concentration.

In this study, we aimed to investigate whether these three radioligands could be used to image high- and low-affinity state receptors of 5-HT_1B_ by evaluating if any correlation can be made between increasing agonistic activity and displacement by increased endogenous 5-HT concentrations.

## Methods

Reference ligand **1** and the *N*-desmethyl precursor for its radiolabeling were synthesized in the Imaging Probe Development Center at NIH. Other reference compounds and precursors were provided by AstraZeneca. All radioactivity measurements were decay-corrected. Group data are mean ± SD.

### Pharmacological assays

*EC*_50_ values and intrinsic activity (antagonist/agonist efficacy) were measured by comparing 5-HT_1B_ receptor ability to bind GTPγ^35^S when stimulated with 5-HT. The agonist/antagonist effect (percentage) was measured at 10 μM and *EC*_50_ in a range from 10 μM to 170 pM. *EC*_50_ was determined as the concentration at half the maximum of the dose-response curve [19].

### Radiochemistry

[^11^C]**1** and [^11^C]**3** were prepared through similar procedures. Thus, [^11^C]methane was obtained through the ^14^N(p,α)^11^C reaction by bombarding a mixture of nitrogen and hydrogen (10%) gas with a beam (35 μA) of protons (16.7 MeV) generated from a biomedical cyclotron (GE PETtrace; Uppsala, Sweden). At the end of irradiation, the target gas was transferred to a Tracerlab synthesis module (GE PETtrace, Uppsala, Sweden) where the [^11^C]methane was trapped by cooling the gas to − 140 °C. After replacing the target gas with helium, the [^11^C]methane was converted into [^11^C]methyl iodide during 5 min of recirculation of radioactive gas through an iodine evaporation oven at 70 °C, an iodination oven at 720 °C, and a [^11^C]methyl iodide trap. The [^11^C]methyl iodide was released by heating the trap to 190 °C and then passed through a heated column of AgOTf to generate [^11^C]methyl triflate. The [^11^C]methyl triflate was transferred to a septum-sealed reaction vessel (5 mL) containing *N*-desmethyl precursor (0.1–0.5 mg) and NaOH (0.5 M, 6 μL) in acetone (400 μL). After 2 min at ambient temperature, the reaction mixture was diluted with water (3 mL) and injected onto a reversed phased ACE C-18 HPLC column (250 × 10 mm; Advanced Chromatography Technologies Ltd.; Aberdeen, UK) using a mobile phase of 60% aqueous HCO_2_NH_4_ (0.1 M) in acetonitrile. Eluate was monitored for absorbance (*λ* = 254 nm) and for radioactivity. The desired fraction (*t*_R_ = 3–3.5 min for [^11^C]**1** or 3.5–4 min for [^11^C]**3**) was collected and diluted with water (30 mL). The radioactive product was trapped on a Sep-pak, washed with water (2 mL), eluted with ethanol (0.5 mL,) and formulated in saline (5.5 mL). Finally, the formulated product was filtered through a sterile filter (0.22 μm; Millipore Millex®GV) into a sterile injection vial.

The radiochemical purity of the product was determined with reversed phase HPLC on an ACE 5 C18-HL column (3.9 × 300 mm, 10 μm particle size; Advanced Chromatography Technologies Ltd), with eluate monitored in a series for absorbance at 254 nm and radioactivity (β-flow detector; Beckman, Fullerton, CA, USA). For the analysis, the column was eluted at 3 mL/min with a gradient of 10–90% MeCN in HCO_2_NH_4_ (0.1 M) for 7 min. The desired radioligand (*t*_R_ = 3.5 min) was identified by its co-mobility with authentic reference ligand.

The molar activity (*A*_m_) of the final product was measured with HPLC under the conditions described above for radiochemical analysis. The absorbance (*λ* = 254 nm) response was pre-calibrated for the mass of a ligand. *A*_m_ was calculated as the radioactivity of the radioligand (GBq) divided by the amount of the associated carrier substance (micromole). Each sample was analyzed three times. The average area under the curve (AUC) for each sample was used to calculate the ratio of AUC to concentration.

Procedures for preparation and analysis of [^11^C]**2** have been described previously [16, 20].

### PET experimental procedure

The PET study in NHPs was approved by the Animal Ethics Committee of the Swedish Animal Welfare Agency (Dnr N185/14) and was performed according to the relevant guidelines of the Karolinska Institutet (“Guidelines for Planning, Conducting, and Documenting Experimental Research” (Dnr 4820/06-600) and “Guide for the Care and Use of Laboratory Animals”).

Three female cynomolgus monkeys were supplied by Astrid Fagraeus Laboratory at the Karolinska Institutet (Solna, Sweden). Anesthesia was initiated via intramuscular injection of ketamine hydrochloride (ca. 10 mg/kg) and after, intubation was maintained by the administration of a mixture of sevoflurane, oxygen, and medical air.

The NHPs were observed continuously during the days of PET measurements. Body temperature was maintained by Bair Hugger-Model 505 (Arizant Healthcare Inc., MN) and monitored with an esophageal thermometer. Heart rate, blood pressure, respiratory rate, and oxygen saturation were continuously monitored throughout the experiments. Fluid balance was maintained by a continuous infusion of saline. The NHP head was fixed in position throughout PET data acquisition as described previously [21]. PET measurements were conducted using a high-resolution research tomograph (Siemens). Radioactivity in the brain was measured continuously for 123 min after radioligand injection according to a preprogrammed series of 34 frames [22].

In an initial PET study to evaluate the specific binding of [^11^C]**3**, one NHP (weight 6.9 kg) was used in a baseline measurement. This monkey was also used in a pretreatment paradigm in which the 5-HT_1B_ antagonist AR-A000002 (AZ10419427; 2.0 mg/kg) was intravenously infused over 6 min starting at 30 min before radioligand injection.

PET measurements using fenfluramine to evaluate the sensitivity of [^11^C]**1** and [^11^C]**3** towards endogenous 5-HT release followed an identical procedure for two NHPs (weight 6.8 and 7.0 kg). Each NHP was used in a baseline and a displacement PET measurement with intravenous infusion of fenfluramine over 5 min starting 15 min after injection of the same radioligand as used in the baseline measurement. The dose of fenfluramine was 1 mg/kg (i.v.) for both radioligands and 5 mg/kg (i.v.) for [^11^C]**3** only. After an unexpected complication, the study was limited to be concluded only at the lower dose level. In summary, results for [^11^C]**1** were acquired from this study (1 mg/kg of fenfluramine) and from Lindberg et al. (5.0 mg/kg of fenfluramine), [15] results for [^11^C]**2** were exclusively acquired from Finnema et al. [16], and all results for [^11^C]**3** were acquired from this study. The experimental procedure in this study was designed to replicate the procedure described by Finnema et al. [16].

### PET data analysis

Average PET images were manually co-registered to a T1-weighted brain magnetic resonance (MR) image previously obtained for each monkey. The co-registration parameters were applied to the dynamic PET data. Regions of interest (ROI) were delineated manually for the whole brain, occipital cortex, globus pallidus, caudate nucleus, putamen, ventral striatum (nucleus accumbens), cerebellum, frontal cortex, midbrain, thalamus, and hippocampus. Regional radioactivity was expressed as standardized uptake value (SUV), which was calculated as uptake (becquerels/milliliter)/injected radioactivity (becquerels) × body weight (grams).

The cerebellum was used as a reference region because this region has negligible density of 5-HT_1B_ receptors [23, 24]. Specific binding to the 5-HT_1B_ receptor was defined as the difference between the total radioactivity concentration in the target brain region and that in the cerebellum, each using the area under the time-radioactivity curve for the scan interval 45–123 min. The binding potential (*BP*_*ND*_) was defined as the ratio of specific binding to the radioactivity concentration in the cerebellum, calculated by simplified reference tissue model (SRTM). The occipital cortex was selected as the ROI to evaluate the sensitivity of radioligands towards changes in synaptic 5-HT concentrations because this sizable region has been shown to have a high density of 5-HT_1B_ receptors.

## Results

**Fig. 2:**
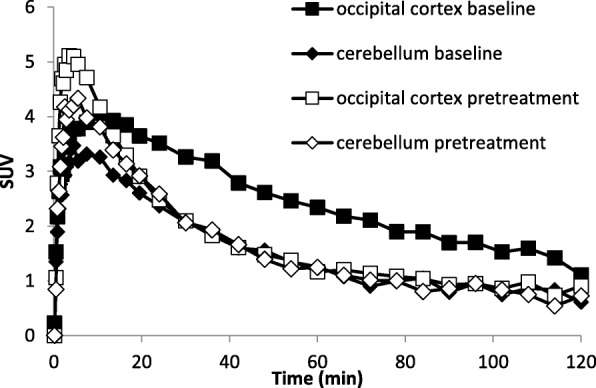
Regional time-activity curves for PET measurements in a cynomolgus monkey after i.v. injection of [^11^C]**3** for the occipital cortex and cerebellum in baseline and pretreatment PET measurements (AR-A000002 2.0 mg/kg)

In the first monkey, [^11^C]**3** (168 MBq, *n* = 1; *A*_m_ = 641 GBq/μmol) was injected in a baseline PET measurement. Radioactivity reached a maximum of 2.5 SUV in the whole brain at 10 min after which radioactivity slowly declined (Fig. 2). Radioactivity levels differed markedly between regions with high 5-HT_1B_ receptor density (occipital cortex and globus pallidus) and that of the cerebellum. All regional time-activity curves declined after the initial peak. The *BP*_ND_ calculated with SRTM was found to be 0.7 in the occipital cortex and 0.48 in the globus pallidus.

In the same monkey, 3 h later, a second PET measurement was performed with [^11^C]**3** (166 MBq; *A*_m_ = 652 GBq/μmol) after pretreatment with AR-A000002 (AZ10419427, 2.0 mg/kg, i.v.). There was a markedly lower radioactivity concentration in all examined regions, except for the cerebellum, than in the baseline experiment (Fig. 2). The *BP*_ND_ in the occipital cortex was 0.07 and 0.23 in globus pallidus, a reduction of 90% and 52%, respectively.

**Fig. 3:**
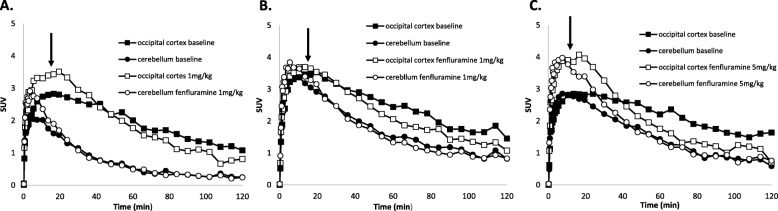
PET measurements of regional time-activity curves for [^11^C]**1** at baseline and in displacement experiments with fenfluramine. **a** For [^11^C]**1** using fenfluramine at 1.0 mg/kg (i.v.) for displacement. **b** For [^11^C]**3** using fenfluramine at 1.0 mg/kg (i.v.) for displacement. **c** For [^11^C]**3** using fenfluramine (5.0 mg/kg, i.v.) for displacement. In all panels, arrows denote injection time of fenfluramine (15 min after radioligand)

In two monkeys, [^11^C]**1** (161 ± 5 MBq, *n* = 4; *A*_m_ = 639 ± 205 GBq/μmol) was injected in a baseline PET measurement followed by a PET measurement 3 h later in which fenfluramine (1.0 mg/kg, i.v.) was administered 15 min after the radioligand. The specific binding in the occipital cortex over the 45- to 123-min period of data acquisition was compared between the two PET measurements. In both monkeys, the calculated decrease was 19% (Fig. 3a). In other regions, the reduction varied from 2% in the midbrain to 33% in the thalamus. Unexpectedly, low displacements of 3 and 9% were observed for globus pallidus.

In two monkeys, [^11^C]**3** (154 ± 6 MBq, *n* = 4; *A*_m_ = 1517 ± 745 GBq/μmol) was injected in a baseline PET measurement followed by a PET measurement 3 h later in which fenfluramine (1.0 mg/kg, i.v.) was administered 15 min after the radioligand. The specific binding in the occipital cortex over the 45- to 123-min period of data acquisition was compared between the two PET measurements. In the two monkeys, the calculated decrease was 16% and 27% (Fig. 3b). In other regions, the change in specific binding varied from + 5% in the midbrain to − 44% in the frontal cortex. In globus pallidus, the decreases were 4 and 11%, respectively.

**Fig. 4:**
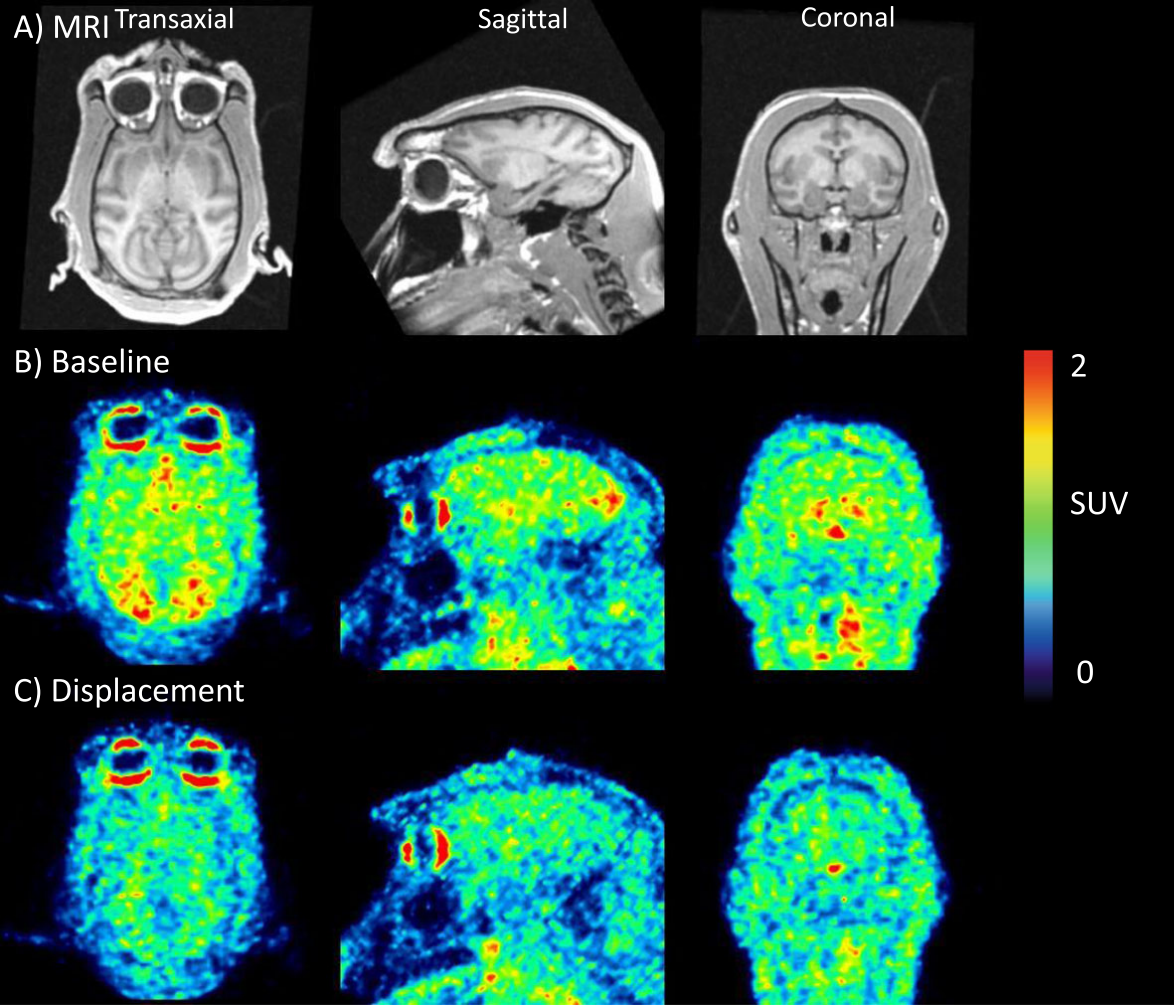
**a** MRI images of a cynomolgus monkey. **b** PET summation images (45–123 min) obtained after i.v. injection of [^11^C]**3** at baseline. **c** PET summation images (45–123 min) obtained after displacement with fenfluramine (5.0 mg/kg, i.v.) injected 15 min after [^11^C]**3**

In two monkeys, [^11^C]**3** (155 ± 6 MBq, *n* = 4; *A*_m_ = 594 ± 43 GBq/μmol) was injected in a baseline PET measurement followed by a PET measurement 3 h later at which fenfluramine (5.0 mg/kg) was administered 15 min after the radioligand (Fig. 4). The mean specific binding in the occipital cortex over the 45- to 123-min period of data acquisition was compared between the two PET measurements. In the monkeys, the calculated decreases were 61% and 62% (Fig. 3c). In other regions, the reduction in specific binding varied from 34% in the hippocampus to 68% in the frontal cortex. Displacements of 35 and 49% were observed for the globus pallidus.

**Fig. 5:**
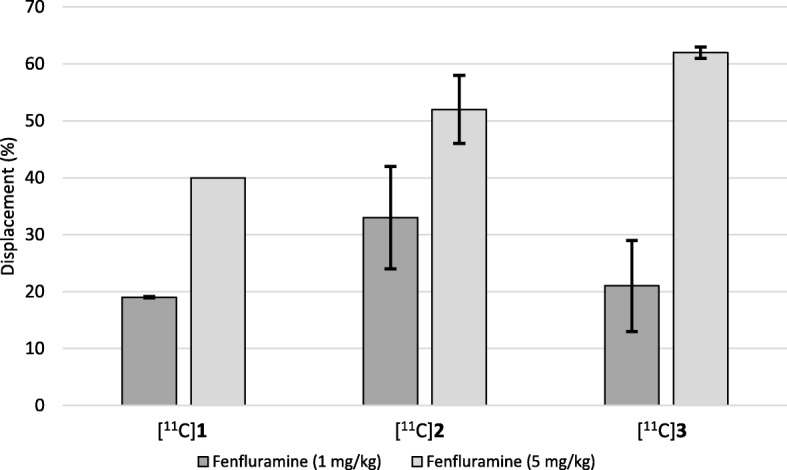
Displacement of radioligand by endogenous 5-HT in percent using fenfluramine (1.0 mg/kg and 5.0 mg/kg). Results for [^11^C]AZ10419096 at fenfluramine (5.0 mg/kg) is based on one monkey [15]. Results for [^11^C]AZ10419369 at both doses are based on three monkeys [16]. All other results are based on two monkeys. Bars show average displacement of radioligand for each dose of fenfluramine and error bars show individual data points where two monkeys were used, and standard deviation were three monkeys were used

Finally, the results of the present study were combined with those from our previously published PET studies with [^11^C]**1** and [^11^C]**2** [15, 16]. In those studies, data for [^11^C]**1** were obtained from one monkey and data for [^11^C]**2** were obtained from three monkeys. After displacement with the higher dose of fenfluramine (5 mg/kg), the sensitivity towards endogenous 5-HT release follows the rank order for antagonist/agonist activity of the three radioligands. This rank order does not appear at the lower dose of fenfluramine (1 mg/kg) (Fig. 5).

## Discussion

We aimed to investigate if PET could be used to image different affinity states of the 5-HT_1B_ receptor. For this purpose, we examined three previously reported PET radioligands having sub-nanomolar affinity and similar log*D* (Fig. 1). [^11^C]**1** is a recently developed full antagonist 5-HT_1B_ PET radioligand [15]. [^11^C]**2** is a mixed efficacy PET radioligand that has been used in multiple studies over the last decade [16, 20]. [^11^C]**3** is an agonist with some antagonist activity that had previously been studied with PET in NHP at baseline only [18]. The pharmacological characterization was performed by AstraZeneca and data only provided as one value for each category. These values should not be viewed as absolute but as presenting relative comparison intrinsic activities of the three radioligands. **1** being the most antagonistic and **3** the least antagonistic.

[^11^C]**3** was used initially in a blocking experiment using the reference 5-HT_1B_ antagonist AR-A000002 (2.0 mg/kg) to confirm specific binding to the 5-HT_1B_ receptor in NHP brain. As for [^11^C]**1** and [^11^C]**2**, the brain radioactivity uptake after the administration of [^11^C]**3** was greatly blocked by AR-A000002 (2.0 mg/kg, i.v.) in all brain regions, in this case by 87–90%. [^11^C]**1** and [^11^C]**3** were examined in displacement studies with intravenously administered fenfluramine (1 mg/kg for both radioligands and 5 mg/kg for [^11^C]**3** only) to induce endogenous 5-HT release. Finally, we compared these results to those previously reported for [^11^C]**1** and [^11^C]**2** at 5 mg/kg [15, 20].

The occipital cortex was used as the primary region of comparison in NHP PET measurement and fenfluramine was used to induce endogenous 5-HT release to increase synaptic 5-HT concentration to displace radioligand bound to 5-HT_1B_ receptors in the brain.

All three radioligands were sensitive to increases in synaptic 5-HT concentration induced with fenfluramine. The response appears to be dose-dependent for each individual radioligand, and displacement was more pronounced with 5 mg/kg fenfluramine than with 1 mg/kg. (Fig. 5). At the higher dose of fenfluramine, the displacement of the agonist [^11^C]**3** was the highest of the three radioligands. The displacement of the antagonist [^11^C]**1** was the lowest at both doses. By themselves, these results are not enough to conclude definitively that an agonist 5-HT_1B_ PET radioligand is more sensitive towards changes in synaptic 5-HT concentrations. The results give some support to the notion that an agonistic 5-HT_1B_ PET radioligand would be more sensitive towards changes in synaptic 5-HT concentrations than an antagonistic radioligand. This has also been demonstrated for the 5-HT_2A_ receptor using PET [25]. Combined PET and microdialysis studies using fenfluramine (5 and 10 mg/kg) have shown that even with a 20- and 35-fold increase in extracellular 5-HT concentration, it was difficult to detect changes in brain 5-HT_1A_ receptor specific binding of [^18^F]MPPF with PET [26]. Here we showed that even with a lower dose of fenfluramine (1 mg/kg), changes in specific binding can be detected for all three 5-HT_1B_ radioligands. Nonetheless, the use of microdialysis could improve a future study because comparing extracellular 5-HT concentrations to changes in specific binding might give less variance than comparing changes in specific binding to dose of fenfluramine.

Another trend is also evident; the specific binding increases with increasing antagonist activity. This follows the idea that an antagonist would have a higher concentration of available receptors to bind to than an agonist, because an antagonist is expected to bind indiscriminately to all affinity states of the receptor. The specific binding (45–123 min) in the occipital cortex calculated for the three radioligands ranges from 0.9 for the agonistic [^11^C]**3** and 1.9 for [^11^C]**2** to 3.2 for the antagonistic [^11^C]**1**.

The apparent challenges in designing a PET study for imaging a high-affinity state receptor are first, to identify suitable PET radioligands with differing intrinsic activity and second, to establish a protocol under which a potential difference in affinity state binding can be appreciated. In this initial study, we have been able to identify some parameters that will be useful for further studies. A dose lower than 1 mg/kg fenfluramine would likely be insufficient to quantify displacement accurately, and a higher dose than 5 mg/kg is not regarded as feasible due to potential risk for the monkeys. More dose levels need to be tested to acquire enough data to make any clear conclusions, and these should be in the range of 1 to 4 mg/kg. The variance between individual monkeys, especially at the lower dose level for [^11^C]**2** and [^11^C]**3**, also suggests that multiple PET measurements for each monkey at each dose would be helpful.

## Conclusion

Three 5-HT_1B_ PET radioligands with high affinity and differing intrinsic activity were identified. They showed dose-dependent displacement by fenfluramine-induced 5-HT release. A dose range of fenfluramine was established within which an extended PET study should take place. The need for multiple PET measurements at each dose for each monkey was also identified. In conclusion, the suggestion for a larger study is to use [^11^C]**1**–**3** in displacement PET measurements as described herein using three separate dose levels of fenfluramine between 1 and 4 mg/kg. Each radioligand should ideally be evaluated more than once at each dose in each monkey.

## Data Availability

The data supporting the conclusions of this article is included within the article.
